# Discovering Ecological Interactions Between Biocontrol Bacterial Strains and Entomopathogenic Nematodes in Button Mushroom Production

**DOI:** 10.3390/microorganisms13030505

**Published:** 2025-02-25

**Authors:** Ivana Potočnik, Ljiljana Šantrić, Jelena Luković, Nikola Grujić, Nikola Anđelković, Ivana Majić, Tanja Drobnjaković, Dejan Marčić, Svetlana Milijašević-Marčić

**Affiliations:** 1 Institute of Pesticides and Environmental Protection, Banatska 31b, 11080 Belgrade-Zemun, Serbia; ivana.potocnik@pesting.org.rs (I.P.); ljiljana.santric@pesting.org.rs (L.Š.); jelena.lukovic@pesting.org.rs (J.L.); tanjadrobnjakovic@gmail.com (T.D.); dejan.marcic@pesting.org.rs (D.M.); 2 Faculty of Agriculture, University of Belgrade, Nemanjina 6, 11080 Belgrade-Zemun, Serbia; grujic@agrif.bg.ac.rs (N.G.); nikola.andj@agrif.bg.ac.rs (N.A.); 3 Faculty of Agrobiotechnical Sciences Osijek, Josip Juraj Strossmayer University of Osijek, Vladimira Preloga 1, 31000 Osijek, Croatia; imajic@fazos.hr

**Keywords:** *Agaricus bisporus*, antagonistic bacteria, *Bacillus amyloliquefaciens*, *Streptomyces flavovirens*, fungus gnat, *Lycoriella ingenua*, entomopathogenic nematodes, *Steinernema feltiae*, compost green mould, *Trichoderma aggressivum*

## Abstract

The substrate for button mushroom (*Agaricus bisporus*) cultivation includes a highly complex microbiome. The aim of the study was to evaluate ecological interactions (synergistic, antagonistic, or additive) between a commercial population of the entomopathogenic nematode *Steinernema feltiae* (EPN) and beneficial microorganisms, bacterium *Bacillus amyloliquefaciens* B-241 (BA) or actinobacterium *Streptomyces flavovirens* A06 (SF). Their relationships were evaluated in efficacy against the pathogenic fungus *Trichoderma aggressivum* and the fungus gnat *Lycoriella ingenua*. Moreover, their impact on mushroom yield was estimated. The synergy factor was calculated as the ratio of observed to expected values regarding their efficacy against *T. aggressivum*/*L. ingenua* and influence on mushroom production. Additive relationships in efficacy against *T. aggressivum* were observed between EPN and BA or SF. As for the impact on yield, synergistic interactions were indicated between each beneficial microorganism and EPN. Considering suppression of *L. ingenua*, a mild antagonistic reaction between EPN and each beneficial microorganism was observed in plots without *T. aggressivum* and additive in plots inoculated with the pathogenic fungus, although high efficacy was achieved in all combinations (>80%). Tested native strains of both beneficial microorganisms could be combined with the commercial EPN strain for successful biological pest and disease control in mushroom production.

## 1. Introduction

The cultivation system of the button mushroom (*Agaricus bisporus* [Lange] Imbach) is very complex. *A. bisporus* is known as a secondary decomposer fungus able to grow on partly decomposed, humic-rich substrates, prepared in a composting process. It is also dependent on the casing layer, which stimulates fructification [1,2]. Substrate preparation is a multistep process guided by a consortium of beneficial microorganisms stimulating fermentation [3]. Organisms inhabiting the same ecological niche enter into diverse relationships, forming numerous associations that can be beneficial (mutualistic), harmful (parasitic and pathogenic), or symbiotic, regardless of whether the relation is between macro- or microorganisms [4,5,6]. A variety of pathogenic microorganisms could be present in these controlled environmental conditions. One of the most devastating species is the pathogenic fungus belonging to the *Trichoderma* genus, the causal agent of compost green mold disease, *Trichoderma aggressivum*. Samuels & W. Gams. As for the mushroom pests, sciarid and phorid flies (Diptera; Sciaridae, Phoridae) are two major groups of insect pests, known to be responsible for crop damage, yield losses, and decrease in quality. Sciarids *Lycoriella* spp. are a common problem in mushroom cultivation during compost phase II (before colonization with mushroom mycelia) so they are further distributed to growers, i.e., mushroom growing houses [7], while phorids *Megaselia* spp. commonly invade compost that is already colonized by the mushroom mycelium [8]. Among them, the sciarid *Lycoriella ingenua* (Dufour) is considered the dominant pest of mushrooms. Its larvae hatch after 21 days and begin feeding on the substrate, mushroom mycelia, and mushroom caps, while phorid larvae ingest mushroom mycelia. Besides causing direct damage to the mushroom crop, the most important impact of sciarid and phorid adults and larvae is the spread of pathogenic fungi [8]. Namely, *L. ingenua* females preferentially lay eggs in areas contaminated with these fungi [9,10]. The flies actively harvest pathogen spores by grooming behavior. Their larvae ingest the spores, which remain viable even after passing through the digestive system, facilitating their spread. Additionally, the presence of the pathogen *T. aggressivum* accelerates sciarid larval development and results in larger adult females, which can be explained by their improved nutrition, pre-digestion, or mushroom defense suppression [8,11].

For decades, mushroom growers have relied on the application of synthetic chemical pesticides (insecticides and fungicides) in an attempt to control mushroom pests and pathogens [12,13]. As a consequence, the harmful effects of chemical pesticides on human health and the environment called for different pest and disease control measures based on biological control agents (BCAs). The limitations of effective use of BCAs are connected to inconsistent or limited control efficacies because of the influence of numerous abiotic and biotic factors (ecological interactions among micro/organisms) [9,13]. Additional drawbacks include challenges of expensive practical in vitro mass production, limited post-application persistence, and narrow specificity of BCAs against target organisms [5]. Therefore, the most success has been achieved in controlled environmental conditions, such as greenhouses and mushroom production facilities.

Regarding biological disease control in button mushrooms, there are several studies on the ability of *Bacillus* species to suppress green mold agents, both in vitro and in vivo [14,15,16,17,18,19]. Additionally, actinobacteria belonging to the genus *Streptomyces* have been reported to not only suppress the negative impact of diseases on the crop but also to boost the yield of edible mushrooms [20]. In an attempt to improve the efficacy of biological control, numerous studies on combining different biocontrol agents for disease management have been conducted. Mixtures of fungal or bacterial antagonists, bacteria and fungi, and/or bacterial and actinobacterial strains were investigated [21,22,23,24,25], but most of them aimed to target only one disease agent. Regarding mushroom pests, studies have primarily focused on entomopathogenic nematodes (EPNs) of the genus *Steinernema* Travassos (Rhabditida: Steinernematidae) [13]. Besides predatory mites, EPNs are the only commercially available biological control agents against mushroom pests and are therefore considered the standard for biological pest management in this crop across Europe [9]. Their infective juveniles play an essential role in killing insects by the toxicity of the gut symbiotic bacteria [26,27]. The efficacy of EPNs is shaped not only by the properties of the soil or substrate they inhabit but also by various biotic interactions [28]. Various biotic factors negatively impact their ability to survive, navigate through the soil, find hosts, and reproduce. The availability of suitable hosts is likely the most critical factor determining the presence and sustainability of EPNs in any given location [29]. Biotic factors also include antibiosis, competition, and the presence of natural enemies such as predators, parasites, and pathogens [30]. Regarding pest control, combined control agents could act autonomously in a host, and the effect on pests is the summary of each one, resulting in an additive effect. The relationship could also be synergistic if the effects of both agents exceed their individual efficacy or antagonistic when agents interfere with each other [31]. Since EPNs rely on their symbiotic bacteria for infection, development, and reproduction, their ability to interact with additional beneficial bacteria in combined treatments depends on complex ecological and biological factors. These include competition for resources, bacterial compatibility, and the influence of co-inoculation on nematode fitness and efficacy in pest suppression [32]. The attempts to combine biocontrol microbial agents could also lead to an incompatibility between the microorganisms by inhibiting each other [22]. Since soil microbiota may affect the efficacy of entomopathogenic nematodes, a clearer knowledge of the microorganisms’ post-application effects on their performance is very important from a practical point of view [28]. Understanding relationships between microorganisms after their introduction in the mushroom cultivation cycle is a way to support strategies aimed at increasing both biological efficiency/quantity and quality of the products. Furthermore, the use of BCAs for pest and disease management is one of the requests of organic agriculture. It also represents an environmentally safe alternative aimed at reducing chemical inputs in integrated agricultural systems [23]. Although there are several in vitro studies regarding the compatibility of EPNs and beneficial bacteria, including *Bacillus* species [33,34], to our best knowledge, there are no published in vivo studies concerning BCAs interactions to fight both pest and disease agents in *Agaricus* production. Therefore, the aim of the study was to evaluate ecological interactions (synergistic, antagonistic, or additive relationships) between the commercial population of EPN *Steinernema feltiae* (Filipjev) and the bacterium *Bacillus amyloliquefaciens* B-241 or actinobacterium *Streptomyces flavovirens* A06 in the management of *T. aggressivum* and *L. ingenua* in edible mushroom production, as well as the impact of co-inoculation of these microorganisms on the total yield of mushrooms.

## 2. Materials and Methods

### 2.1. Pathogenic Fungus and Culture Conditions

The mycopathogen, *Trichoderma aggressivum* f. *europaeum* strain T77 (Accession number: KC555186 in Genbank (https://ncbi.nlm.nih.gov, accessed on 30 January 2013), belongs to the collection of the Institute of Pesticides and Environmental Protection, Belgrade-Zemun, Serbia). The culture was stored in 30% glycerol (Zorka Pharma, Šabac, Serbia) at −80 °C. The inoculum was prepared as previously described by Milijašević-Marčić et al. [25]. Conidia concentration was set to 10^6^ conidia per m^2^ of casing soil and used for artificial inoculation in mushroom growing room experiments.

### 2.2. Biological Control Agents: Beneficial Bacterial Strains and Entomopathogenic Nematodes

Two microorganisms originating from mushroom compost produced in Serbia (Compost Factory Uča d.o.o., Vranovo): actinobacterium *S. flavovirens* strain A06 and bacterium *B. amyloliquefaciens* strain B-241, were used in the study. The actinobacterial and bacterial strains had been previously identified, and their stimulative and/or antifungal traits were confirmed [17,18,20]. The strains were preserved in 20% glycerol at −20 °C in the culture collection of the Institute of Pesticides and Environmental Protection, Belgrade-Zemun, Serbia. The treatments were prepared as described by Milijašević-Marčić et al. [25]. Bacterial and actinobacterial suspensions were adjusted to approximately 10^9^–10^8^ CFU mL^−1^, respectively, and validated by the plate count technique.

The commercial population of EPN *S. feltiae* (Nemaplus, E-nema GmbH, Schwentinental, Germany) was used in the mushroom growing room experiments. Fresh infective juveniles (IJ) were produced in vivo, using the last larval instar of the greater wax moth *Galleria mellonella* (Lepidoptera: *Pyralidae*) [35]. Infective juveniles, not older than 4–5 days with 99% viability (as confirmed on the treatment day), were used at a concentration previously adjusted to 0.75 × 10^6^ IJ m^−2^ in 450 mL of water per m^2^.

### 2.3. Mushroom Growing Room Experiments

**Figure 1:**
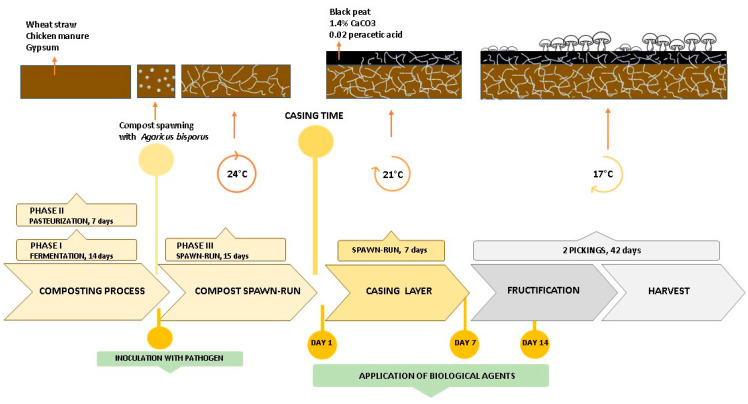
Schematic culture cycle of *Agaricus bisporus*.

Experiments 1 and 2 were conducted in the same manner, placed in an environmentally controlled mushroom growing room at the Institute of Pesticides and Environmental Protection, Belgrade-Zemun, Serbia. The first experiment was conducted during January and February 2024, while the second was performed from March to May 2024. Mature compost phase II, used in the study, was produced in the Compost Factory Uča d.o.o., Vranovo, Serbia, during two phases. In phase I, a mixture of wheat straw, chicken manure, and gypsum was subjected to aerobic fermentation (14 days of outdoor process). In phase II (pasteurization), partially composted substrate was moved into a facility for heat treatment (seven days of controlled indoor process) [16]. Compost spawned with *A. bisporus* mycelium was regarded as phase III compost (Figure 1).

Plastic containers (l × w × h dimensions of 0.285 m × 0.2 m × 0.140 m) were filled with 1.5 kg of compost (phase II) and spawned with 1% mycelium of *A. bisporus* A15 (Sylvan, Dunaharaszti, Hungary, zR1). During Phase III (spawn-run), compost containers were incubated at 24 °C, and relative humidity (RH) ranged from 90 to 95% for 15 days. Compost was subsequently covered with a 1.3 kg (40 mm layer) of black peat-based mushroom casing soil (Terahum d.o.o., Veliko Gradište, Serbia). The casing layer was sterilized by peracetic acid 0.02% (15% Peral S, MidraEko, Belgrade, Serbia) and enriched with 1.4% limestone (Tara Stil d.o.o., Belgrade, Serbia) prior to addition. Casing time was identified as the first day of the cultivation cycle. Air temperature was reduced to 17 °C to provide mushroom fruiting body development after case run incubation of the substrate at 21 °C for 8 days (Figure 1). Half of the experimental plots were inoculated with conidial suspension (10^6^ conidia per m^2^ bed area) of *T. aggressivum* f. *europaeum* strain T77 a day after spawning time.

In order to evaluate ecological interactions (synergistic/antagonistic/additive), treatments were conducted starting from the casing time in three split applications on a weekly basis (Figure 1). The treatments listed in Table 1 were tested on *Trichoderma*-inoculated and uninoculated plots.

Uninoculated (CU) and inoculated (CI) control plots were treated with water. All plots were arranged in a randomized block system with six replicates per treatment and placed into the insect-rearing cages, one plot per cage. Mushroom flies *L. ingenua* were monitored under conditions of natural infestation of compost. Individual and combined treatments were evaluated in terms of their efficacy in controlling *T. aggressivum* f. *europaeum* and *L. ingenua*. The impact of different treatments and/or their combinations on mushroom yield (biological efficiency) was also assessed. Mushrooms were hand-picked during two flushes, subsequently categorized as healthy or diseased, and their weight was measured. The efficacy (E%) of BA, SF, EPN, and their combinations was calculated by Abbott’s formula, E (%) = [(Ic − It)/Ic] × 100, where Ic presents the disease incidence in control, and it presents the disease/pest incidence in treated plots [36,37]. Disease incidence was defined as the percentage of diseased mushrooms out of the total yield. Pest incidence was observed by counting *L. ingenua* adults captured on yellow sticky traps inside each cage. The adults were identified using the identification key given by Menzel & Mohrig [38].

The impact of treatments on mushroom yield (biological efficiency, BE%) was calculated as the ratio of the fresh weight of total mushroom yield (healthy and diseased) and the weight of dry spawned substrate, expressed as percentages [39].

Synergistic, antagonistic, and additive interactions were evaluated for the combination of BA + EPN and SF + EPN. The synergy factor (Sf) was calculated as the ratio of observed to expected effects, by Limpel’s formula: Exp = (X + Y) − (XY)/100, Exp—effect from additive responses of two agents; X and Y—the percentage of effect caused by BA or SF and EPN [40]. Consequently, Sf > 1 designated a synergistic reaction, Sf < 1 an antagonistic reaction, and Sf = 1 indicated an additive reaction.

### 2.4. Statistical Analyses

The data obtained from in vivo experiments in a mushroom cultivation chamber were submitted to a one-way factor analysis of variance (ANOVA). The *Fisher’s* test was used for the mean separation to compare the significance of differences among data on the average efficacy of different single or combined beneficial organisms against the green mold agent (*T. aggressivum* f. *europaeum* T77) and their biological efficiency (influence on yield). The level of significance was evaluated at *p* < 0.05 in all tests [41].

The data transformation formula √(x + 0.1) was applied to normalize and eliminate zero values (number of sciarid flies); the percentage efficacy was transformed with arcsin(x). Data were processed using one-way ANOVA (treatment as a factor); the significance of differences between the means of each treatment compared to the control was analyzed by *Fisher’s* post hoc test (*p* < 0.05) [42].

## 3. Results

### 3.1. Pathogen Control

**Figure 2:**
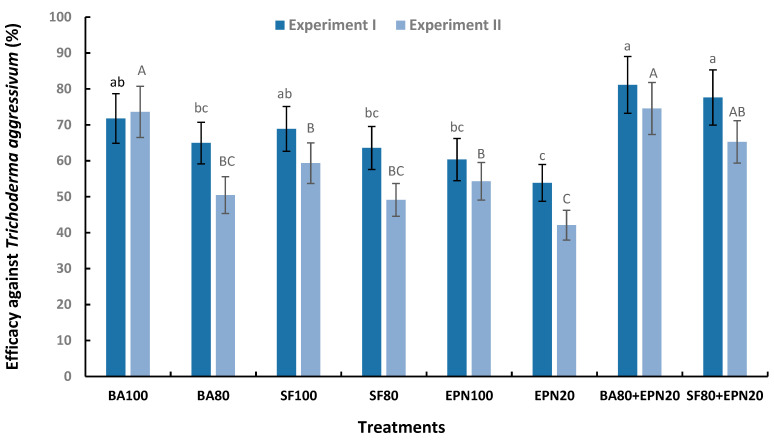
Efficacy (%) of the bacterium *Bacillus amyloliquefaciens* B-241 (BA), actinobacterium *Streptomyces flavovirens* A06 (SF), and entomopathogenic nematode *Steinernema feltiae* (EPN) against *Trichoderma aggressivum* f. *europaeum* T77 on *Agaricus bisporus*; the numbers following abbreviations indicate application rates. Data are means of six replicates ± SE, standard error of means. The same letters (lowercase in experiment I; uppercase in experiment II) designate values that are not significantly different according to *Fisher’s test* (*p* < 0.05).

The first symptoms of green mold disease manifested as brown spots on mushroom caps and stem that appeared on day 17 in both experiments, initially in substrates inoculated with *T. aggressivum* f. *europaeum* T77. Typical green colonies were recorded in inoculated substrates a few days later. The efficacy of BA, SF, and EPN in disease control in both experiments is displayed in Figure 2. The highest efficacy (in experiment I F_7,32_ = 5.75, and experiment II F_7,32_ = 6.94, respectively; *p* < 0.001) was found after the combined applications of BA + EPN (81.13% and 74.55%) and SF + EPN (77.62% and 65.24%). The lowest efficacy was recorded after treatment with EPN (53.85% and 42.08%).

**Figure 3:**
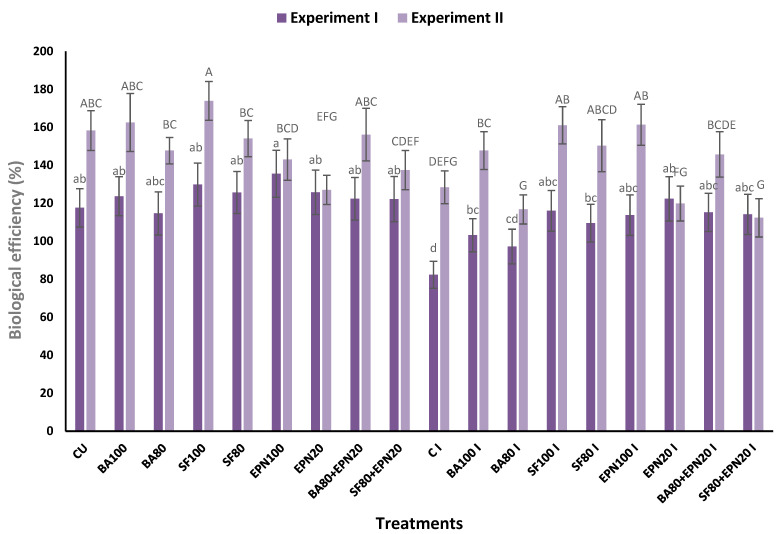
Biological efficiency (%) (impact on mushroom yield) of the bacterium *Bacillus amyloliquefaciens* B-241 (BA100%; BA80%; BA80% + EPN20%), actinobacterium *Streptomyces flavovirens* A06 (SF100%; SF20%; SF80% + EPN20%) and entomopathogenic nematode *Steinernema feltiae* (EPN100%; EPN20%) on *Agaricus bisporus* and control uninoculated (CU) and artificially inoculated with *Trichoderma aggressivum* f. *europaeum* T77 (CI; BA100% I; BA80% I; BA80% + EPN20% I; SF100% I; SF20% I; SF80% + EPN20% I; EPN100 I; EPN20% I); the numbers following abbreviations indicate application rates. Data are means of six replicates ± SE, standard error of means. The same letters (lowercase in experiment I; uppercase in experiment II) designate values that are not significantly different according to *Fisher’s test* (*p* < 0.05).

The impact of three biological agents on mushroom yield (BE, biological efficiency) is presented in Figure 3. Dry spawned substrate weighed 576.7 g in the first experiment and 590.3 g in the second one. Values of biological efficiency ranged from 82.33% to 135.51% in the first experiment and from 112.33% to 173.92% in the second. The highest yield was recorded in treatment with EPN and SF at their standard application rates in both experiments (Figure 3) (F_17,72_ = 2.48 experiment I, F_17,72_ = 4.44 experiment II; *p* < 0.001). Mushroom production varied slightly among all other uninoculated and inoculated treatments in both experiments. The single application of SF at a standard rate enhanced mushroom production by 9.9–10.4% in uninoculated plots, while yield increased by 25.5–40.9% in the presence of the pathogen. The combined application of SF and EPN improved the yield by 3.9% in uninoculated plots and by 38.96% in infected plots compared to the control in the first experiment. In the second experiment, no statistically significant difference in yield was found between control plots and the combination of SF and EPN.

The numbers following abbreviations indicate application rates. Synergy factor (Sf) is a ratio between the observed and expected inhibition. Sf > 1 designates a synergistic reaction, Sf < 1 is an antagonistic reaction, and Sf = 1 indicates an additive reaction. Data are means of six replicates ± SE, standard error of means.

The interaction of combined beneficial organisms in pathogen suppression efficacy (E) and yield impact (BE) was evaluated using their respective synergy factors (Table 2). Additive relationships in terms of efficacy against the pathogen between BA or SF and EPN were determined, based on the synergy factor (Sf) values that approximately equaled 1 in both experiments. Regarding the impact on yield, Sf values between each beneficial bacteria and EPN exceeded 1 in both experiments, revealing synergistic interactions.

### 3.2. Pest Control

**Figure 4:**
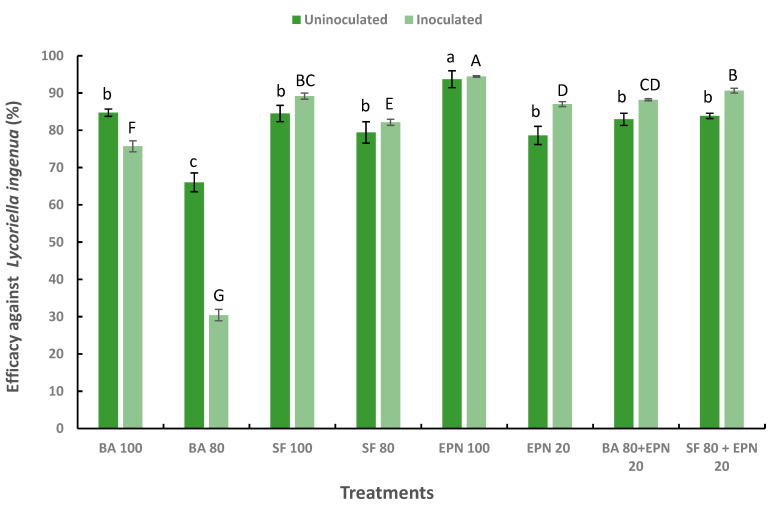
The efficacy (%) of entomopathogenic nematode *Steinernema feltiae* (EPN), bacterium *Bacillus amyloliquefaciens* B-241 (BA), and actinobacterium *Streptomyces flavovirens* A06 (SF) in controlling *Lycoriella ingenua* on white button mushroom (*Agaricus bisporus*) inoculated or uninoculated with *Trichoderma aggressivum* f. *europaeum* T77; the numbers following abbreviations indicate application rates. Data are means of six replicates ± SE, standard error of means. The same letters (lowercase in uninoculated treatments; uppercase in inoculated treatments) designate values that are not significantly different according to *Fisher’s test* (*p* < 0.05).

Considering the scarce number of mushroom flies recorded in all treatments and control plots (due to low natural infestation level) in the first experiment, the data on pest control efficacy are shown only for the second experiment. Regarding pest control, in substrates inoculated with *T. aggressivum* f. *europaeum* T77, Fisher’s post-hoc test showed that the mean number of *L. ingenua* adult flies was significantly lower in treatments in which beneficial organisms were used than in the control (F_8,36_ = 473.02, *p* < 0.001) over the entire test period (Table 3). The lowest mean number of adults was found in the treatment with EPN at the standard application rate, while the highest number was recorded in the treatment with BA at 80%. In addition, the post-hoc test showed that the treatment with EPN 100% had the best overall pest control performance (F_8,36_ = 405.14, *p* < 0.00), achieving an efficacy of over 94% (Figure 4). High pest control efficacy (approximately 90%) was also observed after treatment with SF 100% and a combined application of SF 80% and EPN 20%. A slightly lower efficacy (87–89%) was achieved by treatments with EPN 20% and BA 80% + EPN 20%, while the application of treatments with SF 80% and BA 100% proved to be satisfactory. The lowest efficacy (approximately 30%) was found after the application of BA 80% (Figure 4).

Similar to the findings in inoculated plots, all treatments with beneficial organisms significantly reduced the emergence of *L. ingenua* adults from the mushroom-growing substrate in uninoculated plots compared to the control (F_8,36_ = 60.302, *p* < 0.001) (Table 3). In the inoculated plots, the mean number of total adult flies per treatment per container for the entire test period was significantly lower in cages where EPN 100% was used, whereas the highest adult number was recorded in cages where BA 80% was applied. In addition, Fisher’s test showed that treatment with EPN 100% achieved the highest overall efficacy, of approximately 94% (F_8,36_ = 10.918, *p* < 0.001) (Figure 4). The lowest efficacy (approximately 66%) was recorded after the application of *B. amyloliquefaciens* B-241 80%, while all other applied treatments achieved statistically similar efficacy in controlling pest larvae (78–85%) compared to the control (Figure 4).

The relationship between EPN and either of the beneficial microorganisms BA or SF in *L. ingenua* control was slightly antagonistic in plots uninoculated with *T. aggressivum* as the relevant synergy factor values were 0.90 (BA + EPN) or 0.87 (SF + EPN). Additive relationships were found in the presence of the pathogen as synergy factor values were close to 1 in both combinations of EPN with beneficial microorganisms (Table 4).

## 4. Discussion

Button mushrooms are grown in microbe-rich substrates where a variety of interactions are established among the cultivated fungus and (micro)organisms inhabiting the same niche, ranging from antagonism and competition to symbiosis [43]. In this study, we tried to clarify the complex relationships between different trophic groups of microorganisms and invertebrates in the *Agaricus* production system from a practical point of view. Interactions were evaluated after biocontrol agents were synchronously used to combat one of the most important disease agents, the pathogenic fungus *T. aggressivum* f. *europaeum*, and the fungus gnat (*L. ingenua*) as the most frequent pest. Although investigations on combined applications of BCAs to utilize potential synergistic effects are increasing, most studies have tested pest or disease control possibilities resulting from combined use of two BCAs against a single pest or pathogen [25,29]. However, a small percentage (2%) of recently published research has provided evidence of synergistic effects existing between BCAs after their combined use for disease suppression [44]. Generally, antagonistic interactions were much more common, which is mostly attributed to different biocontrol mechanisms of particular agents [44]. On the other hand, most of the recent studies regarding the combined use of EPNs with entomopathogenic fungi for pest control showed an additive or synergistic effect, while only a few noted an antagonistic effect [31].

In the current study, there were no significant differences in the disease control efficacy between the individual applications of biocontrol agents BA, SF, EPN, and combinations of BA or SF with EPN. Their relationships with EPN regarding pathogen control were designated as additive based on the synergy factor, thus implying that both of these bacteria could be used simultaneously with EPNs in white button mushroom production. In our previous study [25], mutual interactions between BA and SF in the control of *T. agressivum* f. *europaeum*, applied simultaneously as antifungal agents in *A. bisporus* production, were investigated. The study showed that modifying the microbiome of casing soil, even temporarily, by simultaneous application of native microorganisms, resulted in an additive effect regarding disease control and a synergistic effect towards mushroom yield improvement [25]. Recent studies on the impact of *Bacillus velezensis* QST713 (Serenade^®^, Bayer CropSciences, Monheim am Rhein, Germany) on the population dynamics of the microbiota of mushroom compost after artificial inoculation with the green-mould agent showed that the strain QST713 effectively inhibited *T. aggressivum* early on while colonizing the compost [19]. The authors concluded that significant changes in microbial communities occurred after 15 days of *Agaricus* culture when enhanced competition for the same niche between the pathogenic fungus and QST713 strain appears to be involved. On the other hand, Clarke et al. [45] reported that the application of *B. velezensis* QST713 and Kos strains on casing soil did not significantly affect the microbiota composition during the cropping cycle.

It is generally acknowledged that interactions between bacteria and fungi have a remarkable impact on mushroom cultivation. Considering the impact on yield in the current study, the combined application of SF and EPN improved mushroom yield in the first experiment, regardless of inoculation with *T. aggressivum* f. *europaeum*. However, yield in the second experiment was not significantly different in comparison with both *Trichoderma*-inoculated and uninoculated control plots. In addition, synergistic interactions were found between each beneficial microorganism (BA or SF) and EPN. Individual application of EPN led to a 15% yield elevation in uninoculated plots. All three BCAs improved yield more in *Trichoderma* inoculated plots than in uninoculated ones, whether applied individually (at their standard application rates) or combined. The yield improvement was as follows: EPN 30–52%; BA 10–39%; SF 24–31%; EPN + BA 18–37%; EPN + SF 19–21%. Similarly, Pandin et al. [19] reported a yield improvement of 54% when compost inoculated with *T. aggressivum* was treated with the *B. velezensis* strain QST713. In addition, Mumpuni et al. [46] and Potočnik et al. [47] assumed that mushroom pathogens from the genus *Trichoderma* could enhance the growth and fructification of *A. bisporus.* We could speculate that the greater yield promotion in plots or experiments artificially inoculated with *T. aggressivum* was a result of joint actions of BCAs decomposing substrate ingredients and thus providing more nutrients for the *Agaricus* crop.

Regarding pest control, all beneficial organisms applied individually or in combination showed a significantly higher efficacy against the fungus gnat compared to the control, regardless of the presence of the pathogen. The most significant reduction in pest incidence and the highest efficacy (>90%) were achieved by a single treatment with EPN 100%. Treatment with EPN 20% and its combinations with each beneficial microorganism achieved a slightly lower but acceptable efficacy (>80%). Entomopathogenic nematodes have demonstrated their high effectiveness as biocontrol agents against dipteran pests, achieving insect control levels comparable to chemical insecticides [48]. Individual treatments with beneficial microorganisms (BA 100%; SF 80% and 100%) also showed efficacy of >80%. This could be attributed to the entomopathogenic traits of the genus *Streptomyces*. Namely, the actinobacterial strains *Streptomyces griseoplanus* SAI-25, *Streptomyces bacillaris* CAI-155, and *Streptomyces albolongus* BCA-698, isolated from herbal vermi-composts and organically cultivated fields, exhibited a consistent activity against three lepidopteran insects in both laboratory and greenhouse conditions [49]. Treatment with BA 80% proved to be the least successful in suppressing fungus gnat larvae in both inoculated and uninoculated plots. In conclusion, the presence of the pathogen did not influence the efficacy of treatments against the fungus gnat.

Moreover, in vivo experiments closely analogous to commercial production conditions showed 60–100% effectiveness in the control of the fungus gnat when using different commercial populations of *S. feltiae* at application rates of 3 IJs per m^2^ or higher [40,41,42,43,44,45,46,47,48,49,50,51,52]. However, there were also reports showing that *S. feltiae* populations were able to control the fungus gnat with equal effectiveness when drench treatment was applied at much lower rates (0.50–1.5 IJ per m^2^) at casing time [53]. In our current study, a mildly antagonistic relationship between EPN and beneficial microorganisms was found in uninoculated plots regarding the efficacy of pest control, while an additive relationship was found between each of the two in plots inoculated with *T. aggressivum*. Secondary metabolites produced by symbiotic bacteria in *S. feltiae* are diverse and include toxins, enzymes such as lipases and proteases, antibiotics, and lipopolysaccharides. These compounds have been shown to suppress mite populations [54] and inhibit the growth of different microorganisms, such as fungi, Gram-positive bacteria (*Micrococcus, Staphylococcus, Bacillus*), and Gram-negative bacteria [55,56,57]. Additionally, Burns [33] showed that the growth and development of *Steinernema carpocapse* populations were negatively affected in the presence of beneficial strains *Bacillus cereus* BA77 and *Bacillus* sp. E727 in vitro, while other *Bacillus* spp. strains E69 and E61 supported the development of EPN. The author presumed that the development and reproduction of *S. carpocapse* could be suppressed after feeding on these bacteria, although their eggs hatched normally in the presence of the bacteria examined. Grewal and Hand [58] found that *Bacillus* spp. inhibited reproduction of the nematode *Caenorhabditis elegans* (similar to *Steinernema* spp.), while beneficial bacteria of the genera *Pseudomonas, Serratia, Xanthomonas, Acinetobacter*, and *Actinobacter* supported their development and reproduction. Additionally, El-Ashry and El-Marzoky [34] reported that a formulated product based on *Bacillus megaterium* 6% was toxic at the standard application rate to the EPNs *Heterorhabditis bacteriophra* (mortality 41%) and *Steinernema carpocapse* (mortality 50%) under laboratory conditions. In this study, an individual application of *S. feltiae* achieved 94% efficacy in pest control. However, a slight antagonistic interaction was observed when *S. feltiae* was combined with the tested beneficial microorganisms, with efficacy dropping to 84% in uninoculated plots and approximately 90% in pathogen-inoculated plots. In our view, this mild antagonism is unlikely to negatively affect mushroom production when the commercial strain of *S. feltiae* was applied in combination with *B. amyloliquefaciens* B-241 or *S. flavovirens* A06. Given the limited number of recent studies on the use of EPN in *A. bisporus* cultivation, future research should explore the potential of other EPN species and their interactions with beneficial bacteria. Additionally, investigating the role of EPN-derived secondary metabolites in pathogen and pest suppression could provide new insights into their effectiveness as biocontrol agents in mushroom production. Considering the high efficacy of these biocontrol treatments, future research should also focus on economic feasibility, assessing the cost-effectiveness of combining *S. feltiae* with beneficial bacteria in commercial mushroom production. A comprehensive cost-benefit analysis would provide valuable insights into the economic advantages of integrated biological control strategies over conventional pest management approaches.

## 5. Conclusions

Discovering ecological interactions between BCAs in the mushroom cropping cycle is an important step in moving towards ecologically sound pest and disease control approaches. The study showed acceptable levels of efficacy in green mold disease control with no significant differences between treatments (BA or SF and their combinations with EPN). The relationships of BA or SF and EPN in green mold disease control were designated as additive. The highest efficacy in *L. ingenua* control (>90%) was achieved by an individual treatment with EPN. Individual treatments with beneficial microorganisms (BA 100%; SF 80% and 100%) also showed satisfactory efficacy (>80%). Mild antagonistic relationships regarding pest control efficacy were observed between EPN and BA, and between EPN and SF in uninoculated plots, while an additive relationship was detected between EPN and each of the two beneficial microorganisms in plots inoculated with *T. aggressivum*. In addition, synergistic interactions between each beneficial microorganism and EPN were observed regarding mushroom yield. Depending on compost quality, yield was improved regardless of inoculation with *T. aggressivum* f. *europaeum* in the first experiment, while there were no significant differences in mushroom production in the second one. In conclusion, the combined application of SF and EPN provided satisfactory control of both targeted organisms (pathogen and pest) and a yield increase. Besides achieving high efficacy in pest and pathogen control, the BCAs tested in the study demonstrated mutual compatibility in the mushroom production system, thus recommending their synchronous application.

## Figures and Tables

**Table 1:** Treatments evaluated in the mushroom growing room and their abbreviations.

Uninoculated Treatments		Inoculated Treatments	
BA100	*Bacillus amyloliquefaciens* B-241 100% (1 × 10^9^ CFU g^−1^ in 1 L H_2_O per m^2^)	BA100 I	*Bacillus amyloliquefaciens* B-241 100% 1 × 10^9^ CFU g^−1^ in 1 L H_2_O per m^2^) + TA
BA80	*Bacillus amyloliquefaciens* B-241 80% (8 × 10^8^ CFU g^−1^ in 1 L H_2_O per m^2^)	BA80 I	*Bacillus amyloliquefaciens* B-241 80% (8 × 10^8^ CFU g^−1^ in 1 L H_2_O per m^2^) + TA
SF100	*Streptomyces flavovirens* A06 100% (1 × 10^8^ CFU g^−1^ in 1 L H_2_O per m^2^)	SF100 I	*Streptomyces flavovirens* A06 100% (1 × 10^8^ CFU g^−1^ in 1 L H_2_O per m^2^) + TA
SF80	*Streptomyces flavovirens* A06 80% (8 × 10^7^ CFU g^−1^ in 1 L H_2_O per m^2^)	SF80 I	*Streptomyces flavovirens* A06 80% (8 × 10^7^ CFU g^−1^ in 1 L H_2_O per m^2^) + TA
EPN100	*Steinernema feltiae* 100% (0.75 × 10^6^ IJs in 450 mL H_2_O per m^2^)	EPN100 I	*Steinernema feltiae* 100% (0.75 × 10^6^ IJs in 450 mL H_2_O per m^2^) + TA
EPN20	*Steinernema feltiae* 20% (0.15 × 10^6^ IJs in 450 mL H_2_O per m^2^)	EPN20 I	*Steinernema feltiae* 20% (0.15 × 10^6^ IJs in 450 mL H_2_O per m^2^) + TA
BA80 + EPN20	*Bacillus amyloliquefaciens* B-241 80% (8 × 10^8^ CFU g^−1^ in 1 L H_2_O per m^2^) + *Steinernema feltiae* 20% (0.15 × 10^6^ IJs in 450 mL H_2_O per m^2^)	BA80 + EPN 20 I	*Bacillus amyloliquefaciens* B-241 80% (8 × 10^8^ CFU g^−1^ in 1 L H_2_O per m^2^) + *Steinernema feltiae* 20% (0.15 × 10^6^ IJs in 450 mL H_2_O per m^2^) + TA
SF80 + EPN20	*Streptomyces flavovirens* A06 80% (8 × 10^7^ CFU g^−1^ in 1 L H_2_O per m^2^) + *Steinernema feltiae* 20% (0.15 × 10^6^ IJs in 450 mL H_2_O per m^2^)	SF80 + EPN 20 I	*Streptomyces flavovirens* A06 80% (8 × 10^7^ CFU g^−1^ in 1 L H_2_O per m^2^) + *Steinernema feltiae* 20% (0.15 × 10^6^ IJs in 450 mL H_2_O per m^2^) + TA
CU	Control uninoculated	CI	Control inoculated with TA
		TA = inoculation with *Trichoderma aggressivum* f. *europaeum* T77	

**Table 2:** Interactions between the microorganisms *Bacillus amyloliquefaciens* B-241 (BA) or *Streptomyces flavovirens* A06 (SF) and entomopathogenic nematode *Steinernema feltiae* (EPN) in terms of efficacy in control of *Trichoderma aggressivum* f. *europaeum* T77 and impact on mushroom productivity.

Treatment	Exp.	Biological Efficiency (BE, %) Impact on Mushroom Yield			Efficacy (E, %) in Suppression of Green Mould Disease Agent		
		Observed BE	Expected BE	Synergy Factor (Confidence Interval)	Observed E	Expected E	Synergy Factor (Confidence Interval)
		Mean ± SE			Mean ± SE		
BA 80 + EPN 20	I	122.35 ± 9.05	96.23	1.27 (1.24–1.40)	81.13 ± 7.89	83.72	0.97 (0.89–1.07)
	II	156.12 ± 13.86	92.39	1.69 (1.58–1.91)	74.55 ± 7.23	71.31	1.04 (0.98–1.06)
SF 80 + EPN 20	I	122.18 ± 3.40	93.39	1.31 (1.20–1.34)	77.62 ± 7.68	83.09	0.94 (0.85–1.04)
	II	137.44 ± 10.36	85.38	1.61 (1.38–1.93)	65.24 ± 5.89	70.53	0.92 (0.86–1.01)

**Table 3:** The impact of entomopathogenic nematode *Steinernema feltiae* (EPN), bacterium *Bacillus amyloliquefaciens* B-241 (BA), and actinobacterium *Streptomyces flavovirens* A06 (SF) on the survival of *Lycoriella ingenua* adults (mean ± SE) per treatment per container inoculated or uninoculated with *T. aggressivum* f. *europaeum* T77; the numbers following abbreviations indicate application rates.

Treatments	Number of *Lycoriella ingenua* Adults	
	Inoculated	Uninoculated
BA 100	49.8 ± 3.0 c	10.6 ± 0.7 c
BA 80	142.6 ± 1.3 b	22.6 ± 1.5 b
SF 100	22.2 ± 0.4 ef	10.2 ± 1.4 c
SF 80	36.6 ± 1.3 d	13.6 ± 1.7 c
EPN 100	11.4 ± 0.4 g	4.2 ± 1.6 d
EPN 20	26.6 ± 1.3 e	14.0 ± 1.5 c
BA 80 + EPN 20	24.2 ± 1.4 e	11.3 ± 1.0 c
SF 80 + EPN 20	19.2 ± 0.6 f	11.2 ± 0.5 c
Control	205.0 ± 9.9 a	69.4 ± 1.9 a

**Table 4:** Interaction between the beneficial microorganisms *Bacillus amyloliquefaciens* B-241 (BA) or *Streptomyces flavovirens* A06 (SF) and entomopathogenic nematode (EPN) *Steinernema felitae* in terms of efficacy against the mushroom fly *Lycoriella ingenua* in uninoculated plots or those inoculated with the mycopathogen *Trichoderma aggressivum* f. *europaeum* T77; the numbers following abbreviations indicate application rates.

Treatment	Inoculation with Mycopathogen	Efficacy (E) in Controlling *Lycoriella ingenua*		
		Observed E	Expected E	Synergy Factor (Confidence Interval)
		Mean ± SE		
BA 80 + EPN 20	Uninoculated	83.86 ± 11.2	93.43	0.90 (0.87–0.94)
	Inoculated	88.19 ± 24.2	90.97	0.97 (0.95–0.99)
SF 80 + EPN 20	Uninoculated	83.86 ± 11.2	96.04	0.87 (0.85–0.90)
	Inoculated	90.63 ± 19.2	97.68	0.93 (0.92–0.93)

Synergy factor (Sf) is a ratio between the observed and expected inhibition. Sf > 1 designates a synergistic reaction, Sf < 1 an antagonistic reaction, and Sf = 1 indicates an additive reaction. Data are means of six replicates ± SE, standard error of means.

## Data Availability

The original contributions presented in this study are included in the article. Further inquiries can be directed to the corresponding author.
